# Optimizing Photosensitizer Delivery for Effective Photodynamic Inactivation of *Klebsiella pneumoniae* Under Lung Surfactant Conditions

**DOI:** 10.3390/pathogens14070618

**Published:** 2025-06-21

**Authors:** Fernanda Alves, Isabelle Almeida de Lima, Lorraine Gabriele Fiuza, Zoe A. Arnaut, Natalia Mayumi Inada, Vanderlei Salvador Bagnato

**Affiliations:** 1 São Carlos Institute of Physics (IFSC), University of São Paulo (USP), São Carlos 13566-590, Brazil; isabelle.almeida016@gmail.com (I.A.d.L.); lorraine.fiuza@ifsc.usp.br (L.G.F.); natalia.inada@ifsc.usp.br (N.M.I.); vander@ifsc.usp.br (V.S.B.); 2 Department of Biomedical Engineering, Texas A&M University, College Station, TX 77843, USA; 3 CQC-IMS, Chemistry Department, University of Coimbra, 3004-535 Coimbra, Portugal; zoearnaut@gmail.com

**Keywords:** *Klebsiella pneumoniae*, lung surfactant, antimicrobial photodynamic therapy, indocyanine green, copolymers

## Abstract

*Klebsiella pneumoniae* is a Gram-negative, encapsulated bacterium recognized by the World Health Organization (WHO) as a critical priority for new therapeutic strategies due to its increasing multidrug resistance (MDR). Antimicrobial photodynamic therapy (aPDT) has emerged as a promising alternative to antibiotics, exhibiting a broad spectrum of action and multiple molecular targets, and has been proposed for the treatment of clinically relevant infections such as pneumonia. However, despite excellent in vitro photodynamic inactivation outcomes, the success of in vivo therapy still faces challenges, particularly due to the presence of lung surfactant (LS) in the alveoli. LS entraps photosensitizers, preventing these molecules from reaching microbial targets. This study investigated the potential of indocyanine green (ICG) in combination with the biocompatible polymer Gantrez™ AN-139 for the photoinactivation of *K. pneumoniae*. Initial in vitro experiments demonstrated that aPDT with ICG alone is effective against *K. pneumoniae* in a concentration- and light dose-dependent manner, achieving total eradication at 75 µg/mL of ICG and 150 J/cm^2^ of 808 nm light. When aPDT was performed with similar parameters in the presence of LS, no bacterial killing was observed. However, a significant synergistic effect was observed when ICG (25 µg/mL) was combined with a low concentration of Gantrez™ AN-139 (0.5% *m*/*v*) in the presence of dipalmitoylphosphatidylcholine (DPPC), the main component of LS. This formulation resulted in a substantial reduction (3.6 log_10_) in *K. pneumoniae* viability. These findings highlight the potential of Gantrez™ AN-139 as an efficient carrier to enhance the efficacy of ICG-mediated aPDT against *K. pneumoniae*, even in the presence of lung surfactant, a necessary step before the in vivo experiments.

## 1. Introduction

Over the past two decades, pneumonia has remained a major global health concern. In 2021 alone, it accounted for approximately 2.2 million deaths worldwide. In the United States, about 1.5 million adults are diagnosed with pneumonia annually, resulting in nearly 1 million hospitalizations and 50,000 deaths. Pneumonia can be classified into community-acquired and hospital-acquired infections, which differ significantly in etiology and resistance profiles. Community-acquired pneumonia is often caused by pathogens such as *Streptococcus pneumoniae*, for which hospitalization rates declined by 23% between 2002 and 2011, likely reflecting the success of vaccination programs. By contrast, hospital-acquired pneumonia, particularly ventilator-associated pneumonia, is frequently associated with multidrug-resistant organisms. Pneumonia caused by *Klebsiella* spp., a pathogen commonly linked to nosocomial infections, showed a 35% increase in hospitalizations during the same period, highlighting its rising clinical relevance and resistance potential [1]. *Klebsiella pneumoniae* is an encapsulated Gram-negative bacterium belonging to the *Enterobacteriaceae* family and is included among the “ESKAPE” pathogens, which are designated by the World Health Organization (WHO) as priority targets for the development of new therapeutic strategies. As an opportunistic pathogen, *K. pneumoniae* ranks among the leading causes of nosocomial infections, urinary tract infections, and bloodstream infections, among others. It exhibits a high potential for developing multidrug resistance (MDR), including the production of extended-spectrum β-lactamases (ESBLs) and various carbapenemases such as KPC (Klebsiella pneumoniae carbapenemase), NDM (New Delhi metallo-β-lactamase), OXA-48, and VIM (Verona integron-encoded metallo-β-lactamase), which significantly compromise the effectiveness of antibiotics, representing one of the greatest global public health threats of the 21st century [2,3,4].

Antimicrobial photodynamic therapy (aPDT) has emerged as a promising alternative to antibiotics due to its broad-spectrum activity and multiple molecular targets, which hinder the development of microbial resistance. In addition to being effective against resistant microorganisms, studies indicate that aPDT can enhance bacterial susceptibility to antibiotic therapy [5,6,7]. The mechanism of action of aPDT involves the activation of a photosensitizer (PS) by light at a specific wavelength. Upon reaching an excited energy state through electron transition, the PS can react with organic substrates that in turn interact with molecular oxygen, generating reactive oxygen species (ROS) such as superoxide anion (O_2_^•–^), hydroxyl radical (^•^OH), and hydrogen peroxide (H_2_O_2_) (type I reaction). Alternatively, it may transfer energy directly to ground-state oxygen, forming singlet oxygen (^1^O_2_) (type II reaction) [8,9]. Given the short lifespan of these reactive species and their localized generation only at irradiated sites, aPDT also offers the advantage of minimal systemic toxicity [10,11].

Several in vitro studies have evaluated the efficacy of aPDT against *K. pneumoniae*, utilizing various photosensitizers and light parameters to achieve significant bacterial inactivation. For instance, a study demonstrated that methylene blue (MB), at a concentration of 100 μg/mL and after activation by red light at 665 nm (25 J/cm^2^), resulted in a 7 log_10_ reduction in bacterial count [12]. Additionally, another study tested 5-aminolevulinic acid (5-ALA) and methyl aminolevulinate (MAL), both at 10 mM concentrations and after activation by white LED light (400–780 nm) with a fluence of 120 J/cm^2^, resulting in 3.2 log_10_ and 4.3 log_10_ reductions, respectively, in extended-spectrum β-lactamase (ESBL)-producing *K. pneumoniae* [13]. Building on these findings, the present research group has focused in recent years on developing a safe and effective photodynamic inactivation protocol targeting pneumonia using indocyanine green (ICG). This photosensitizer, approved by the U.S. Food and Drug Administration (FDA), has been widely employed in medical diagnostics and therapeutic contexts, demonstrating promising outcomes in photodynmaic therapy for both oncological applications and microbial inactivation [14,15,16,17]. One particular advantage of ICG is the use of near-infrared illumination, which enables deeper tissue penetration and facilitates access to internal organs in a less invasive manner [18,19]. In this sense, the treatment of patients with pneumonia by aPDT would be performed with external illumination.

**Figure 1:**
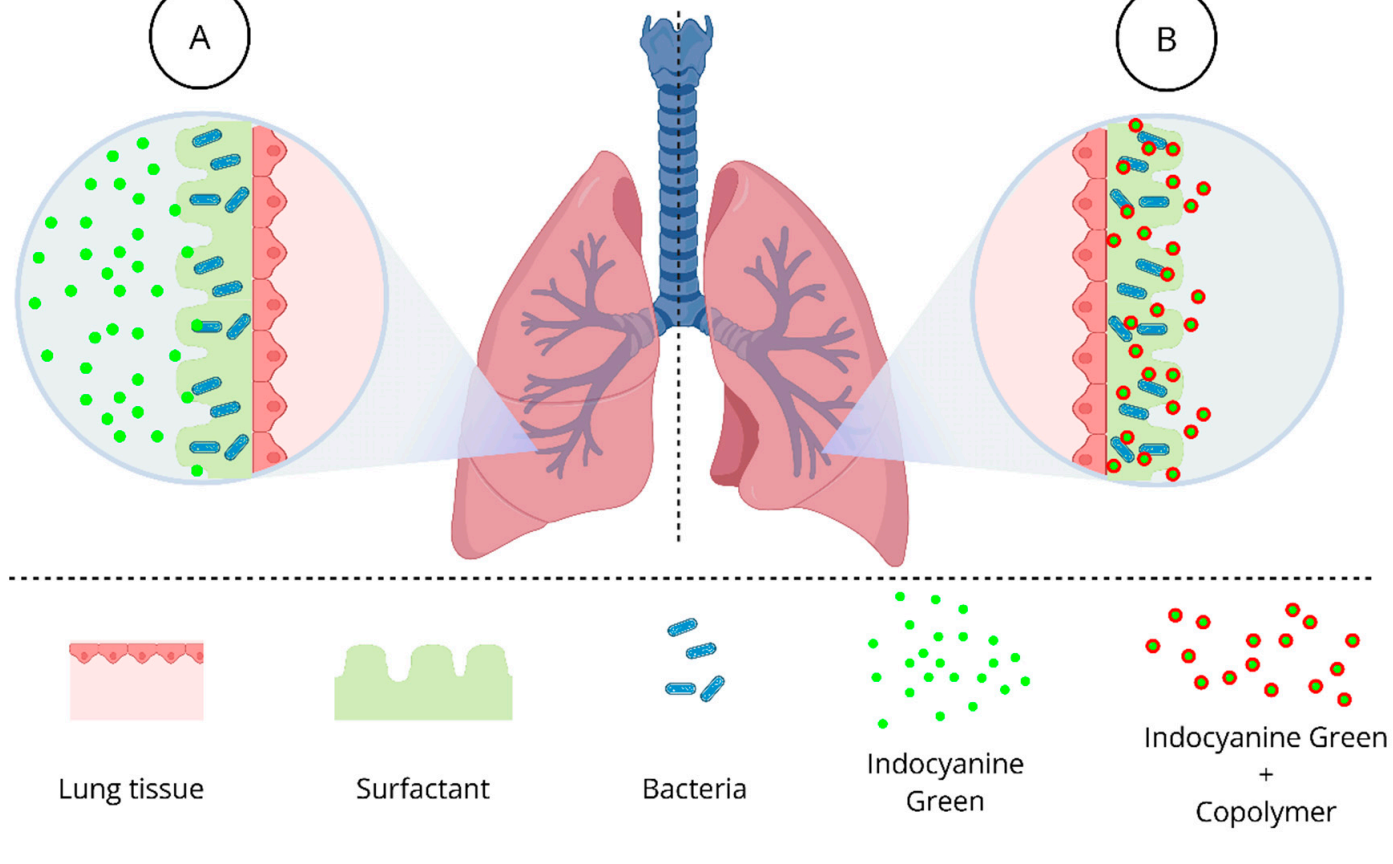
Despite excellent in vitro photodynamic inactivation outcomes, the success of in vivo therapy still faces challenges due to the presence of lung surfactant (LS). LS entraps photosensitizers, such as indocyanine green, preventing these molecules from reaching microbial targets (**A**). A promising strategy is the combination of ICG with Gantrez™ AN-139, a polyvinyl methyl ether/maleic anhydride copolymer (PVM/MA), which enables controlled dissolution and targeted release of ICG, resulting in significant microbial inactivation (**B**).

However, despite excellent in vitro photodynamic inactivation outcomes and demonstrated delivery of both the PS and light in animal models [20,21,22], the success of in vivo therapy still faces challenges, particularly due to the presence of lung surfactant (LS). LS entraps photosensitizers, preventing these molecules from reaching microbial targets [23], as shown in Figure 1A. Lung surfactant is a complex mixture that lines the entire alveolar epithelium and is mainly composed of phospholipids and proteins. It plays a crucial role in mammalian respiration by reducing surface tension and preventing alveolar collapse during exhalation. Additionally, it serves as a protective barrier between the bloodstream and external air [24,25]. Previously, our research group investigated various approaches to enhance the efficacy of aPDT against *S. pneumoniae*, particularly addressing the challenge posed by LS. These strategies evaluated the use of emulsifiers, perfluorocarbon, oxygen nanobubbles, and copolymers. The most promising results were obtained by combining ICG with Gantrez™ AN-139, a polyvinyl methyl ether/maleic anhydride copolymer (PVM/MA). This formulation demonstrated significant microbial inactivation while maintaining safety for human lung epithelial (A549) and fibroblast (MRC-9) cell lines [26]. These in vitro findings suggest that the ICG-Gantrez™ AN-139 combination effectively overcomes the limitations imposed by LS, offering new perspectives for the treatment of infections caused by *K. pneumoniae*.

The formulation of ICG with Gantrez likely relies on noncovalent electrostatic interactions, particularly hydrogen bonding and ionic interactions between the sulfonate groups of ICG and the carboxylic groups generated upon Gantrez hydrolysis [27]. These interactions can prevent dye aggregation and shield it from environmental quenching factors, while also maintaining the fluorescence profile critical for in vivo imaging. Additionally, Gantrez exhibits intrinsic antimicrobial and bioadhesive properties, which can synergize with the photodynamic effect of ICG, especially in biofilm settings such as infected wounds or pulmonary infections. In formulations, Gantrez enables controlled dissolution and targeted release of ICG (Figure 1B), with potential for dual-action therapy: mucoadhesion for retention at the site of interest and light-triggered reactive oxygen species (ROS) generation for microbial inactivation [28].

Despite the promising in vitro results of ICG-mediated aPDT against *S. pneumoniae* and the demonstrated ability of the ICG–Gantrez™ formulation to overcome the LS barrier, its effectiveness against *K. pneumoniae*—a Gram-negative bacterium classified as a critical priority pathogen in pneumonia—remains uninvestigated. Therefore, this study aimed to evaluate the efficacy of ICG–Gantrez™ AN-139-mediated aPDT against *K. pneumoniae* in the presence of lung surfactant, addressing one of the key obstacles to its clinical application in pulmonary infections.

## 2. Methodology

### 2.1. Photosensitizer Formulation and Light Source

The indocyanine green (ICG—Ophtalmos, São Paulo, Brazil) was used as a photosensitizer in the present work. ICG was weighed and diluted in Milli-Q water to obtain the stock solution (1 mg/mL) prior to each experiment. This stock was diluted at 465 µg/mL, and the working solutions were prepared from this in the experiments. The polymer Gantrez AN-139™ (Ashland, Wilmington, DE, USA) was prepared by diluting the powder in Milli-Q water (30% *w*/*v*), followed by vigorous mixing and heating in an oven at 95 °C to promote gel formation. The formulations were prepared by diluting the gel stock solution in Milli-Q water together with the ICG solution. Samples were centrifuged at 4400 rpm for 5 min to ensure complete homogenization and removal of bubbles. For irradiation, an LED system developed at the São Carlos Institute of Physics, University of São Paulo (IFSC—USP), was used, with a peak emission at 808 nm and an irradiance of 162 mW/cm^2^. Light doses ranging from 100 to 250 J/cm^2^ were applied.

### 2.2. Strain and Growth Conditions

The Gram-negative bacterium *K. pneumoniae* from American Type Culture Collection^®^ (ATCC 13883) was used in the present study. The strain was stored in tryptic soy broth (TSB) with 20% (*v*/*v*) glycerol at −80 °C. For the assays, the bacterium was first inoculated onto a brain heart infusion (BHI) agar plate, and then incubated for 18 h at 37 °C. From this plate, 3–5 colonies were scraped and added to 10 mL of BHI broth, followed by incubation for 18 h. The inoculum was then prepared by diluting 500 µL of this pre-inoculum in 9.5 mL of BHI broth and incubating for 3 h, until the bacteria reached the mid-log phase of growth. After this period, the bacterial suspension concentration was adjusted to 10^7^ cells/mL in PBS by measuring the optical density at 600 nm in a spectrophotometer.

### 2.3. Antimicrobial Photodynamic Therapy

First, the efficacy of ICG-mediated aPDT was evaluated. For this, 450 µL of PBS, 25 µL of the bacterial suspension, and 25 µL of ICG (final concentration ranging from 25 to 125 µg/mL) were added to a 24-well plate, and the samples were incubated in the dark for 30 min. After this period, the samples were irradiated at 808 nm with light doses of 100 to 250 J/cm^2^. Additional samples received only ICG, only light, or no treatment (control group). It is important to emphasize that all aPDT parameters and the methodology used in this study were based on and developed from our previous work [26], in which ICG-mediated aPDT was evaluated against *S. pneumoniae* (ATCC^®^ 49619™, 5 × 10^7^ CFU). In that study, the combination of 10 µM ICG with 0.2% (*w*/*v*) of the polymer, following 40 min of incubation in the dark, proved highly effective for photosensitizer delivery and resulted in complete eradication of the bacterial load. Additionally, a pilot assay using *K. pneumoniae* was conducted to provide an initial screening and assess the general response trend of this strain.

Then, to evaluate the inhibitory effect of LS on aPDT, its main component, dipalmitoylphosphatidylcholine (DPPC, Chem-Impex, Wood Dale, IL, USA), was prepared in PBS at a concentration of 30 mg/mL. After that, 25 µL of this solution was added to the plate containing the bacterial suspensions, and then the best aPDT protocol obtained in the previous test was performed with the same parameters to verify and compare the effect of the LS on aPDT.

Finally, the use of the polyvinyl methyl ether/maleic anhydride copolymer (PVM/MA) Gantrez™ AN-139 in combination with ICG was evaluated as a strategy to overcome the limitations imposed by LS (DPPC) on aPDT. It is important to emphasize that previous studies showed that Gantrez™ exhibits antimicrobial effects by itself; for this reason, initially, a range of Gantrez™ concentrations were evaluated (from 0.5 to 2.6% (*w*/*v*)), and the concentration that did not show an antimicrobial effect was selected to be used in combination with aPDT. The treatment protocol then followed the same parameters as previously described.

### 2.4. Evaluation of the Treatments

After treatments, serial dilutions (10^−1^ to 10^−5^) were performed for each sample using PBS. Then, 10 μL of each dilution was plated in duplicate on BHI plates, and the plates were incubated for 24 h. After this period, the colonies were manually and visually counted, the average between the duplicates of each sample was obtained, the number of colony forming units per milliliter (CFU/mL) was calculated, and graphs were plotted using Origin 2022b.

### 2.5. Statistical Analysis

CFU/mL values were log-transformed prior to statistical analysis. Subsequent statistical analysis was performed using one-way ANOVA to determine if there were significant differences among groups. Tukey’s honestly significant difference (HSD) test was employed for pairwise comparisons at an alpha level of 0.05.

## 3. Results and Discussion

**Figure 2:**
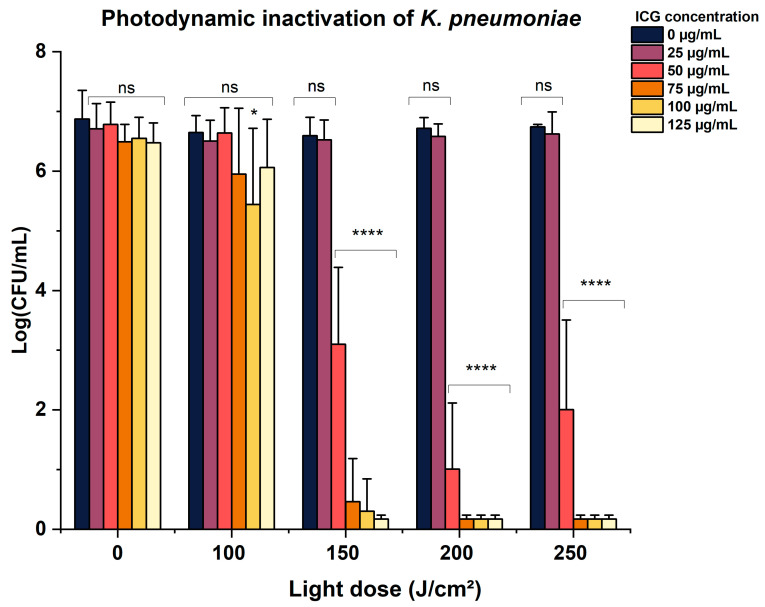
Mean and standard deviation values of log_10_(CFU/mL) of *K. pneumoniae* suspensions after aPDT using different ICG concentrations combined with a range of light doses (*n* = 6). * *p* < 0.05; **** *p* < 0.0001; ns: not significant (*p* > 0.05).

Photodynamic inactivation of Gram-negative bacteria is a challenge due to the structure and complexity of their cell wall, which consists of two membranes. The outermost membrane is rich in lipopolysaccharides (LPSs), which not only hinders the uptake of the photosensitizer but also drastically reduces the interaction of the cell wall with anionic molecules, such as ICG [29,30]. Thus, to enable the use of this PS, the aPDT parameters were extensively explored, with concentrations ranging from 25 to 125 µg/mL and light doses from 100 to 250 J/cm^2^ being evaluated. The inactivation results are presented in Figure 2.

According to Figure 2, the photodynamic inactivation was concentration- and dose-dependent, with microbial reductions occurring at concentrations starting at 50 µg/mL and 150 J/cm^2^ (*p* < 0.0001), and complete elimination achieved with 75 µg/mL. Notably, both the applied light dose (up to 200 J/cm^2^) and ICG concentration (up to 100 µg/mL) fall within ranges considered acceptable for translational applications. Additionally, neither the PS at different concentrations nor the light alone caused microbial reduction, indicating that all observed inactivation was due to the photodynamic effect. These findings agree with studies by Liu et al. [13], who, in order to inactivate 3 log_10_ of *K. pneumoniae* using 5-ALA, a precursor of an anionic PS, had to employ high concentrations (10 mM, 1311 µg/mL) and light doses (120 J/cm^2^). Similarly, Huang et al. [31] and Qingxiang et al. [32] achieved a reduction in this bacterium that was greater than 4 log_10_ using Photofrin, also an anionic PS, by combining it with potassium iodide (KI).

To the best of our knowledge, this is the first study to report on the use of a PS that absorbs in the near-infrared region for the inactivation of *K. pneumoniae*. This is particularly important for potential applications in treating pulmonary infections, such as pneumonia, as this wavelength allows for greater tissue penetration, enabling external light delivery [21]. Previous studies conducted by our research group have evaluated ICG-mediated aPDT against *S. pneumoniae*, and the results obtained in the present work are in line with the previously published data. In 2017, we reported an in vitro inactivation of *S. pneumoniae* by aPDT, with a combination of light at 850 nm and 10 μM ICG [22]. Moreover, the effect of aPDT on alveolar macrophages, which are key phagocytes in the innate immune defense of the lung, was also evaluated. Importantly, alveolar macrophage viability was above 90% following aPDT with ICG and light at 850 nm, suggesting that ICG-mediated aPDT is effective and does not harm the host immune system. Although ROS levels were not directly measured in the present study, previous investigations involving aPDT with ICG against *Pseudomonas aeruginosa* [33], *Staphylococcus aureus*, *Escherichia coli*, and *Candida albicans* [34] have consistently demonstrated ROS generation. Therefore, the observed antibacterial effect can reasonably be attributed to ROS-mediated mechanisms.

However, although light delivery and PS activation are crucial for effective aPDT, the complexity of the pulmonary microenvironment must be considered for successful therapy. The presence of LS and its main component, dipalmitoylphosphatidylcholine (DPPC), has been shown to decrease the antimicrobial effectiveness of ICG-mediated aPDT against *S. pneumoniae* [26].

**Figure 3:**
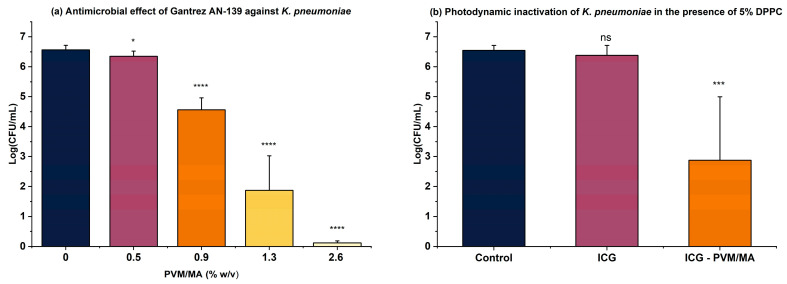
(**a**) Antimicrobial effect of Gantrez AN-139 at different concentrations after irradiation (150 J/cm^2^); (**b**) aPDT using 25 µg/mL of ICG + 0.5% (*w*/*v*) Gantrez AN-139 in a medium containing 5% (*v*/*v*) dipalmitoylphosphatidylcholine (DPPC) (150 J/cm^2^) (*n* = 4). * *p* < 0.05; *** *p* < 0.001; **** *p* < 0.0001; ns: not significant (*p* > 0.05).

In this regard, the polymer Gantrez AN-139 has proven to be an efficient ICG carrier, enabling the inactivation of over 6 log_10_ of *S. pneumoniae* even in the presence of LS [26]. Thus, in the present study, the proof of concept was extended to *K. pneumoniae*, where the antimicrobial activity of the polymer was first evaluated at different concentrations. As illustrated in Figure 3a, toxicity begins at a concentration of 0.9% (*w*/*v*), so 0.5% (*w*/*v*) was selected for combining with ICG. Figure 3b shows that aPDT with ICG alone at 25 µg/mL had no effect. However, when combined with the polymer, a significant microbial reduction (3.6 log_10_) was observed, supporting previous findings using *S. pneumoniae*, where Gantrez AN-139 acted synergistically with ICG, effectively overcoming the LS barrier.

Polymers of various types have been widely used in the literature as delivery vehicles, as they can enhance the bactericidal action of drugs as well as their targeting and penetration into pathogen membranes. Moreover, these components can improve the photostability, bioavailability, and solubility of PSs [35], as reported by Caffarel-Salvador et al. [28], who proposed a delivery system using Gantrez AN-139 to enhance methylene blue delivery in skin wounds. Following the same principle, Requena et al. [36] used this polymer to improve ALA permeation in tumors in mice, assuring the safety of using the polymer. Regarding Gantrez AN-139 and ICG, the use of this formulation poses some advantages that can potentiate the PS delivery and overcome the limitations related to ICG. Indocyanine green, a near-infrared fluorophore widely used for medical diagnostics and optical imaging, suffers from pronounced aqueous instability due to aggregation and photodegradation, especially at the high concentrations required for in vivo applications [27]. In this context, Gantrez^®^ emerges as a compelling candidate due to its amphiphilic, mucoadhesive properties and biocompatibility. When used in microneedle platforms or nanoparticulate formulations, Gantrez not only stabilizes encapsulated drugs like rifampicin but also enhances residence time in mucosal tissues, increases bioavailability, and facilitates translocation across mucosal barriers. These properties suggest that Gantrez–ICG combinations could extend ICG’s fluorescence lifetime and biodistribution in pulmonary applications [28,37]. The way that Gantrez protects the photosensitizer from surfactant is probably by promoting a solution that protects the molecules from the electrostatic charge of the surfactant.

Another important characteristic of a drug delivery vehicle is its mucoadhesive properties. The pulmonary mucus barrier plays a dual role in lung physiology, offering protection against pathogens while simultaneously posing a challenge to drug delivery systems. Its viscoelastic and adhesive nature—composed mainly of mucin fibers, lipids, and cellular debris—hinders the diffusion and penetration of therapeutic agents, especially macromolecules and nanoparticles [28,38]. In diseases such as cystic fibrosis, chronic obstructive pulmonary disease, or pneumonia, the thickening of mucus and the formation of biofilms exacerbate this issue by creating heterogeneous microenvironments that inactivate potential drugs [37,39]. Although topical pulmonary administration is highly desirable because it reduces systemic side effects, enhancing molecule retention at the treatment site and preventing rapid elimination from the lungs due to ciliary movement requires that the polymer used possesses such properties [40,41]. Gantrez AN-139, in addition to exhibiting high mucoadhesiveness, is also biodegradable and biocompatible, making it a promising candidate for pulmonary application [42,43].

The interaction between ICG and Gantrez™ AN-139 presents a promising avenue for investigation. Future studies are needed to determine whether Gantrez enhances ICG uptake, stabilizes the dye, or modulates local ROS diffusion dynamics. Nevertheless, hypotheses can be formulated based on the existing literature. The association between ICG and Gantrez is likely driven by noncovalent electrostatic interactions, particularly hydrogen bonding and ionic interactions between the sulfonate groups of ICG and the carboxylic groups formed upon Gantrez [27]. These interactions may prevent ICG aggregation and protect it from environmental quenching, thereby preserving its fluorescence properties, an essential feature for in vivo imaging. Moreover, Gantrez possesses inherent antimicrobial and bioadhesive properties, which could act synergistically with ICG’s photodynamic effect, particularly in biofilm-associated infections such as those found in wounds or the respiratory tract. In a formulation, Gantrez allows for the controlled dissolution and targeted release of ICG, offering the potential for a dual-action therapeutic approach: mucoadhesion for prolonged retention at the infection site, and light-activated ROS generation for effective microbial inactivation [28].

Finally, it is important to mention that given the presence of commensal microbiota in the lungs, the effects of aPDT on commensal microbiota should be considered. While aPDT is designed to be locally activated and exert its effects selectively at the site of illumination, it is not inherently species-specific. Therefore, beneficial microbes in the irradiated area may also be affected, particularly those embedded in the same biofilm or exposed to generated ROS. Future studies should investigate the impact of aPDT on the pulmonary microbiome to evaluate potential dysbiosis and guide strategies for preserving microbial balance during treatment. Another aspect that should be considered when applying this formulation in in vivo and clinical studies is its pharmacokinetics. In vivo applications of ICG are supported by extensive pharmacokinetic data extracted from a clinical study: the dye is almost entirely cleared by the liver, with ~80% excreted unchanged in bile within 18 h, and has a biphasic plasma half-life of 3–4 min (fast phase) and ~70–90 min (terminal phase) [44]. Regarding Gantrez™ AN-139, although specific studies are lacking, this polymeric excipient belongs to the PVM/MA class, which is biodegradable and has been used safely in microneedles and hydrogels. It degrades via hydrolysis into small, excretable fragments and has demonstrated biocompatibility in multiple formulations [45].

## 4. Conclusions

This study demonstrates that while indocyanine green (ICG)-mediated antimicrobial photodynamic therapy (aPDT) is highly effective against *Klebsiella pneumoniae* under controlled in vitro conditions, its efficacy is significantly hindered in the presence of lung surfactant (LS), which simulates the pulmonary environment. The incorporation of Gantrez™ AN-139, a biocompatible polymer with mucoadhesive and antimicrobial properties, into the ICG formulation successfully restored the antimicrobial activity of aPDT, even in the presence of dipalmitoylphosphatidylcholine (DPPC), the main component of LS. The combination of 25 µg/mL of ICG with 0.5% (*w*/*v*) Gantrez resulted in a significant reduction (3.6 log) in bacterial viability, highlighting the polymer’s potential as an effective delivery system to overcome the surfactant barrier. These findings reinforce the potential of ICG–Gantrez™ AN-139 as a promising strategy for the topical photodynamic treatment of *K. pneumoniae*-related lung infections, offering a viable alternative to conventional antibiotics and contributing to the development of new therapeutic tools against multidrug-resistant pathogens.

## Data Availability

The original contributions presented in this study are included in the article. Further inquiries can be directed to the corresponding author.
